# Numerical and Experimental Study on Mixing Performances of Simple and Vortex Micro T-Mixers

**DOI:** 10.3390/mi9050204

**Published:** 2018-04-27

**Authors:** Mubashshir Ahmad Ansari, Kwang-Yong Kim, Sun Min Kim

**Affiliations:** 1 Department of Mechanical Engineering, Zakir Husain College of Engineering and Technology, Aligarh Muslim University, Aligarh 202001, India; mub.ansari@yahoo.com; 2 Department of Mechanical Engineering, Inha University, Incheon 22212, Korea; 3 WCSL of Integrated Human Airway-on-a-Chip, Inha University, Incheon 22212, Korea

**Keywords:** micromixer, vortex micro T-mixer, planar serpentine microchannel, microfluidics

## Abstract

Vortex flow increases the interface area of fluid streams by stretching along with providing continuous stirring action to the fluids in micromixers. In this study, experimental and numerical analyses on a design of micromixer that creates vortex flow were carried out, and the mixing performance was compared with a simple micro T-mixer. In the vortex micro T-mixer, the height of the inlet channels is half of the height of the main mixing channel. The inlet channel connects to the main mixing channel (micromixer) at the one end at an offset position in a fashion that creates vortex flow. In the simple micro T-mixer, the height of the inlet channels is equal to the height of the channel after connection (main mixing channel). Mixing of fluids and flow field have been analyzed for Reynolds numbers in a range from 1–80. The study has been further extended to planar serpentine microchannels, which were combined with a simple and a vortex T-junction, to evaluate and verify their mixing performances. The mixing performance of the vortex T-mixer is higher than the simple T-mixer and significantly increases with the Reynolds number. The design is promising for efficiently increasing mixing simply at the T-junction and can be applied to all micromixers.

## 1. Introduction

Recently, microfluidic devices have gained popularity for the development of miniaturized analysis systems with wider applications like chemical, biochemical reactions, biomedical devices and drug delivery [1,2,3,4,5,6,7,8,9,10,11,12,13]. Manipulating microparticles/cells and small volume of fluids has lead us to the development of microdevices with specific functionalities, like separation/sorting and mixing of fluids. Testing or analysis of microfluidics systems can be performed with reduced cost and time. Rapid mixing of fluids is an essential process for the homogenization of the samples in these microfluidics devices. Recently, numerous research works have been carried out on the design and development of new micromixers. Micromixers can be broadly categorized into two groups: active and passive micromixers [14,15]. Active micromixers require some external source of energy or any moving parts for stimulating the flow and are generally more efficient in mixing than passive micromixers. However, they are difficult to fabricate and integrate with the main microfluidic systems. Passive micromixers avoid such problems by just including geometrical variations in the microchannel to manipulate the laminar flow (uniaxial flow) for mixing of fluids.

There are numerous designs of passive micromixers reported in the literature, working on different mixing principles. The key idea of enhancing the mixing rate is increasing the area of the interface of the fluid streams and decreasing the length of diffusion. Effective micromixers have the capability of increasing the interface area by adopting certain techniques. Chaotic micromixer using staggered herringbone grooves on the wall showed high effectiveness by exponentially increasing the interface area of the fluid streams [16]. Three-dimensional serpentine [17], curved [18,19,20] and zig-zag [21,22] microchannels have been demonstrated in mixing of fluids. The obstacles in the microchannel have been reported to increase the mixing of fluids by creating advection mixing. Liu et al. fabricated specific bas-relief microstructures on the floor of the microchannel using a direct printing process for mixing [23]. The other technique to increase the interface area of the fluid streams is the multi-lamination of fluid streams by repeatedly splitting and recombining the channels [24,25]. This technique utilizes the two mixing mechanisms: decreasing the length of diffusion and increasing the interface area of fluid streams.

The basic layout of all micromixers can be divided into four separate major units: inlet channels, T- or Y-shaped junctions, mixing channels and outlet channels. Fluid streams are brought into contact by inlet channels connected to a micromixer. Mixing slowly starts right after the fluid streams come into contact at the junction, while major mixing takes place in the mixing channel. Most researchers have focused on designing the mixing channel to create specific flow patterns to enhance mixing. The T- or Y-shaped channel itself is also the simplest T-mixer [26,27,28,29,30,31,32,33,34,35,36,37]. For example, the simple T-mixer is suited for studying the basic concept on the mechanism of the mixing of fluids.

In T-mixers, researchers have identified three different flow regimes developed at different Reynolds numbers and their influences on mixing [26,27,28,29,30,31,32,33,34,35]. These three flow regimes are: stratified, vortex and engulfment flows. In the stratified flow regime, the streamlines of the incoming fluid streams smoothly make a turn and flow along the wall of the microchannel at the bend of the junction forming the symmetric interface in the middle of the main microchannel. In such a situation, mixing is mainly governed by diffusion of molecules across the interface of the fluid streams. The mixing performance is poor because diffusion is a very slow process. At some higher Reynolds numbers (Re ≥ 136) [36,37], the vortex flow starts with the formation of the two vortices, and mixing performance is improved due to the increase in the interface area of the fluid streams. The third and the most important regime for attaining effective mixing is the engulfment flow where streamlines intertwine with each other. In this flow regime, the last two physical phenomena contribute to mixing: an increase in the interface area and a decrease in the diffusion length due to the intertwining of the streamlines. Such flow structures are desired to attain rapid mixing. However, such a flow regime starts at a quite high Reynolds number (~200), which would require high pumping power. Furthermore, the fluid streams have less residence time in the channel for proper mixing.

From the above discussions, it becomes obvious that for the range of Reynolds number (Re ≤ 136), the fluid streams from the inlet channels make a clear, vertical, thin and straight interface across which mixing takes place. The interface gradually becomes broader as the fluid flows towards the other end of the microchannel. Some efforts have to be done for design modification of a simple T-mixer for an early development of the mixing, inducing complex flows at lower Reynolds numbers. An effort was made for the modification to the simple T-mixer with the objective of enhancing the mixing of fluids named as the vortex micro T-mixer [38].

The vortex micro T-mixer shows the formation of vortex flows at low Reynolds numbers [38]. The generated vortex flow stretches the interface of the fluid streams, which increases the mixing of fluids. In this paper, we present a comparative analysis of simple and vortex micro T-mixers at different Reynolds numbers (1–80), both experimentally and numerically. The study has been further extended to demonstrate the mixing performances of novel designs: planar serpentine microchannels combined with the simple and the vortex T-junctions. The design of the vortex T-mixer was proven to be very promising and hence can be selected for integration with all possible current and future micromixer designs.

## 2. Physical and Numerical Model

**Figure 1:**
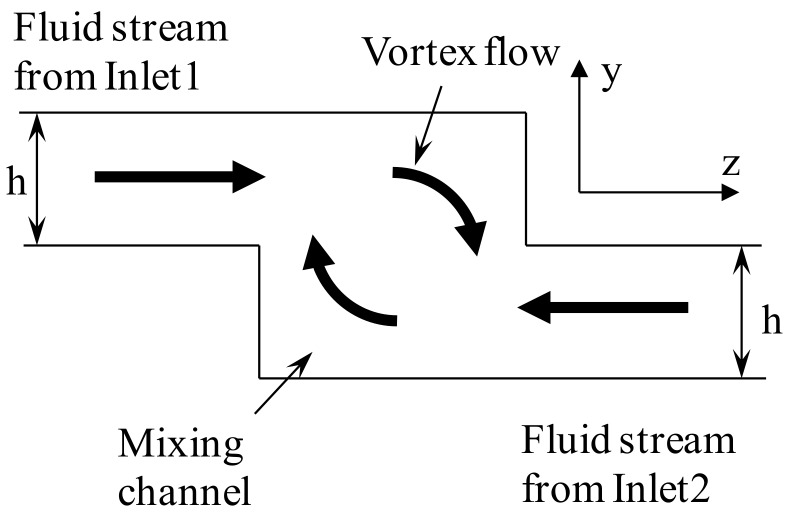
Basic schematic of the idea of vortex micro T-mixer. The two inlet channels of the vortex micro T-mixer are joining the mixing channel at an offset position. The height of the inlet channels is half the height of the mixing channel (h = H/2). The two fluid streams from Inlet 1 and Inlet 2 enter the channel at an offset position and create a vortex flow.

**Figure 2:**
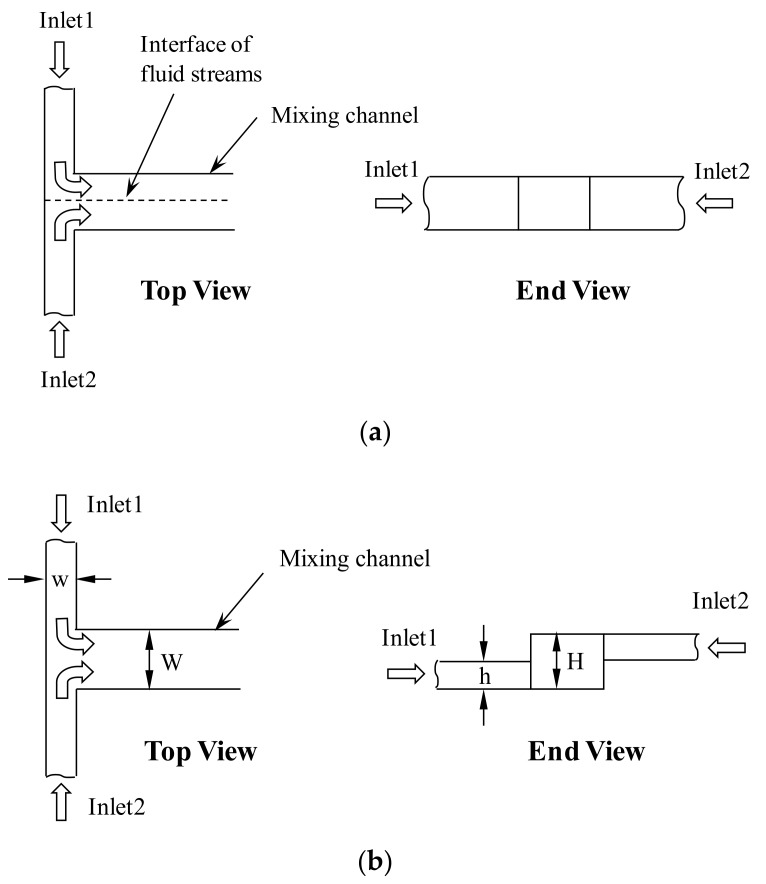
Schematic of the basic idea of the micromixer: (**a**) simple T-mixer and (**b**) vortex micro T-mixer.

The basic concept of increasing the mixing of fluids has utilized the vortex flow formed by the fluid streams flowing through non-aligned inlet channels into the mixing channel (See Figure 1). Schematics of the physical models of simple and vortex micro T-mixers are shown in Figure 2. The width (200 µm), height (200 µm) and length (5 mm) of the main microchannel are equal and fixed for both the vortex and simple T-mixers. The main focus of the present micromixer study is at the junction of the inlet channels and the mixing channel. For the simple T-mixer, the height of the inlet channels is the same as the height of the mixing channel, while in case of the vortex T-mixer, the height of the inlet channels is half the main microchannel (h = H/2). The cross-sectional area of the inlet channels was kept constant and equal for both the vortex (100 µm × 100 µm) and the simple T-mixers (50 µm × 200 µm) in order to compare the mixing performances at similar operating conditions. The length of the inlet channels for both the simple and vortex T-mixers was 1000 µm.

The numerical study on the simple and vortex T-mixers has been carried out at different Reynolds numbers, in a range of 1–80. The effects of the two types of T-junction on the mixing performance have been also investigated by integrating them with a planar serpentine microchannel. The Reynolds number has been calculated considering the characteristic dimensions of the main microchannel with the properties of water.

Numerical simulation on both the simple and vortex T-mixers has been carried out by solving continuity, Navier–Stokes and convection-diffusion equations for the mixing of fluids using a finite volume solver, ANSYS CFX 15.0 (ANSYS, Inc., Canonsburg, PA, USA) [39]. The flows calculated in this work were assumed steady and incompressible. The boundary conditions were normal velocity components at the inlets and zero average static pressure at the outlet. The walls were assigned the no-slip boundary condition. Water and ethanol were selected as the two working fluids with all properties taken at 20 °C. The solution was considered to have attained convergence for a root-mean-square value of 10^−6^ for both the mass fraction and momentum.

Structured grids were applied to discretize the computational domain. In order to capture the complex flow field of vortex flow and the deformation of the interfaces of the fluid streams, the sizes of the computational cells were kept small (~4 µm) near the junction. However, downstream of the T-junction, the flow structure becomes simple in the straight microchannel. Therefore, in this zone, the mesh density was kept low, and hence, the cells were not cubical, rather aligned in the direction of the flow.

The mixing performance of the device was evaluated by calculating the mixing index. It is based on the variance of the mass fraction from the mean concentration. The variance was calculated on the plane perpendicular to the *x*-axis. The values of the mass fraction at equally-spaced sample points on the plane were evaluated to calculate the variance. The mixing index has been defined as:

(1) $$M=1-\sqrt{\frac{\sigma^{2}}{\sigma_{max}^{2}}}$$

whereas $\sigma^{2}$ is the variance of the mass fraction of the fluid component of the mixture and $\sigma_{max}^{2}$ is the maximum variance.

(2) $$\sigma^{2}=\frac{1}{N}\sum {\left(c_{i}-{\bar{c}}_{m}\right)}^{2}$$

The values of the mass fraction have been evaluated at *N* sampling points on the plane. $c_{i}$ is the mass fraction at sampling point *i*, and ${\bar{c}}_{m}$ is the optimal mixing mass fraction. The value of optimal mass fraction ${\bar{c}}_{m}$ is 0.5 for completely mixed fluids.

## 3. Device Fabrication and Experiment

### 3.1. Fabrication

**Figure 3:**
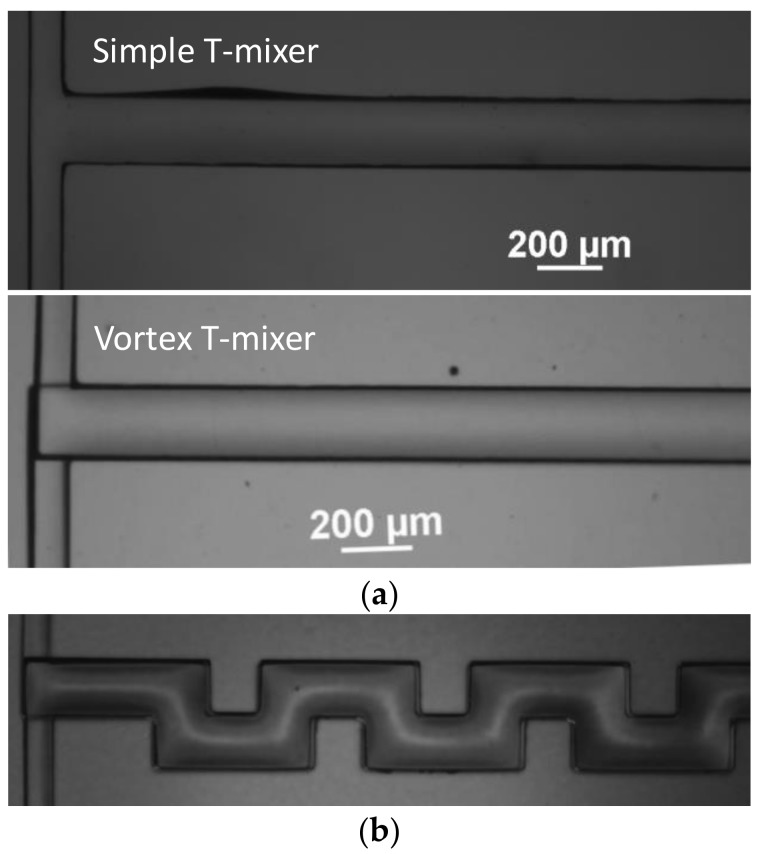
Micrographs of the experimental devices: (**a**) simple and vortex micro T-mixers and (**b**) planar serpentine channel integrated with vortex T-junction.

The micromixer was fabricated in PDMS using the soft lithography technique. The polydimethylsiloxane (PDMS) is a silicon-based polymer (colorless, viscous liquid) widely used for fabricating microfluidic devices. Since the fabrication technique using soft lithography is standardized and well known, the various steps are briefly explained here. The replica process used in the present work is described in detail in previous papers [38,40]. SU-8 photoresist (GM1075, Gersteltec Sarl, Pully, Switzerland) was spin coated on a 4-inch silicon wafer using a spin coater to obtain a 100 µm-thick layer. Soft and hard baking was performed on a hot plate. The coated Si-wafer was exposed to UV light using a standard UV aligner through a high resolution dark film mask followed by post baking. The master mold was developed using SU-8 developer by taking off the unexposed SU-8. The device was fabricated by pouring silicon elastomer and curing agent in a 10:1 weight ratio into the patterned wafer followed by degassing in vacuum desiccators (H42050, Bel-Art product, Wayne, NJ, USA) and heat treatment at 72 °C for 2 h in the convection oven (NDO-400, Sanyo, Osaka, Japan). The device for making the vortex T-mixer was fabricated into two layers, while for the simple T-mixer, into a single layer. The two PDMS layers were bonded together by a manual alignment. Ethanol was applied on the PDMS surface of each layer after corona treatment to preserve the bonding properties of the surface for a sufficiently long time required for manual alignment. The bonded device was kept in the oven at 70 °C for 2 h for heat treatment. The single-layered device was bonded to a glass slide after plasma treatment irreversible sealing. Holes were punched into the PDMS to make the inlet and outlet port using a punch (33-31AA-P, Miltex, Plainsboro, NJ, USA). Figure 3 shows the micrograph of the simple T-mixer, the vortex T-mixer and the planar serpentine channel integrated with the vortex T-junction.

### 3.2. Mixing Experiment

A florescent solution was prepared using distilled water (Milli Q purified) and Rhodamine B (Sigma-Aldrich, St. Louis, MO, USA) with a 100 µM concentration. The Rhodamine B was perfectly dissolved in the water using a vortex stirrer (KMC-1300V, Vision Scientific Co., Ltd., Kyeonggi-do, Korea) and ultrasonic waves. It was ensured that the solution was free of any Rhodamine particle, as this may cause coherence in the microchannel, which may locally disorder the distribution of the florescent intensity and hence may influence the results of mixing. The diffusion coefficients of Rhodamine B in water and ethanol are 2.8 × 10^−10^ m^2^·s^−1^ and 1.2 × 10^−10^ m^2^·s^−1^, respectively, while its molecular weight is 479.17 g/mol [41,42,43,44]. Mixing experiments were carried out using a florescent water solution and pure water on an inverted microscope (Ti-u, Nikon, Tokyo, Japan). Planar mixing images were captured using a charge-coupled device (CCD) camera (DS-Qi1Mc, Nikon, Tokyo‎, Japan). The cross-sectional images were captured using a confocal microscope (LSM10META, Carl Zeiss, Oberkochen, Germany). Fluids were fed into the micromixer by a multi-feed pump (Model 200, KDScience Inc., Holliston, MA, USA) using two syringes through inlet holes connected with Teflon tubes. A constant flow rate of both fluids was maintained in the micromixer for the corresponding Reynolds numbers.

## 4. Results and Discussion

### 4.1. Simple and Vortex Micro T-Mixers

An experimental and a numerical study have been carried out on the simple and vortex micro T-mixer for a wide range of Reynolds numbers (1–80). Their mixing performances have been compared for the same operating conditions. In the numerical study, a preliminary test for grid independency has been performed in order to ensure a mesh-independent solution. Five grid systems have been selected with the number of nodes ranging from 3 × 10^5^–2.2 × 10^6^. Finally, a grid system with the number of nodes 1.26 × 10^6^ was selected for efficiently carrying out further simulations. The mixing index was evaluated at a fixed location (x = 4.5 mm) in the microchannel at Re = 80 for each grid. There was a small variation (0.91%) in the mixing index for the two grids, i.e., 5.8 × 10^5^ and 9.1 × 10^5^. The relative change in the mixing index between the grid systems having 5.8 × 10^5^ and 9.1 × 10^5^ nodes was 0.73%. The difference was found to be of a similar order (0.78%) as the difference between the grid systems with node numbers 1.26 × 10^6^ and 2.21 × 10^6^. However, considering the change in the number of nodes, the latter relative change in mixing is quite low. Based on this result of the grid test, with the number of nodes 1.26 × 10^6^, the numerical results were expected to have minimum error. The simulation results also have numerical diffusion, which can be minimized by increasing the mesh density [45,46]. Ansari et al. [45] reported such an analysis on the presence of numerical diffusion by showing the mass fraction distribution for different mesh densities.

**Figure 4:**
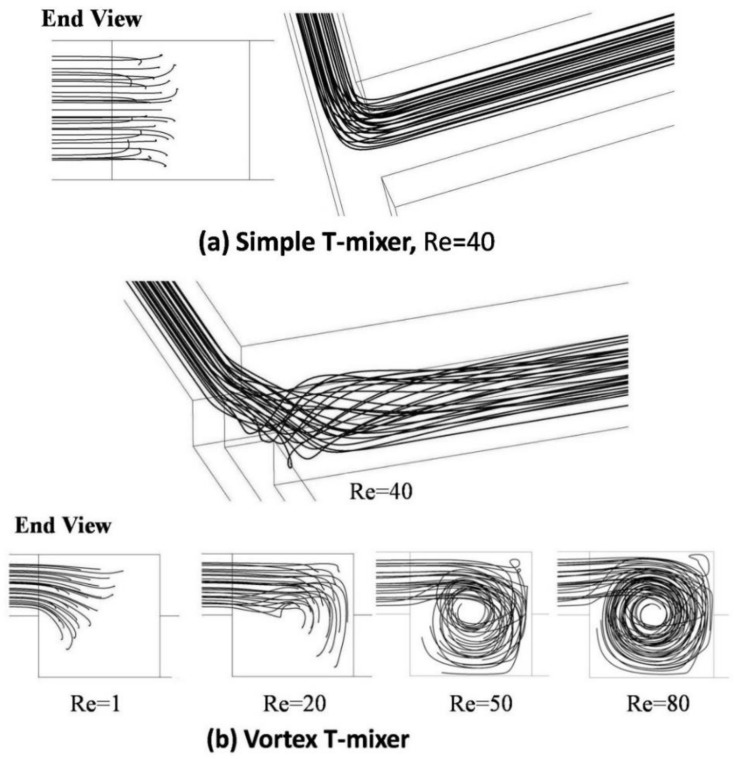
Projected streamlines shown in end view and three-dimensional view: (**a**) simple micro T-mixer and (**b**) vortex micro T-mixer.

Figure 4 shows the streamlines for fluid flow from Inlet 1 for the simple and vortex T-mixers. In the simple T-mixer, the projected streamlines are shown for Re = 40, which represents the development of symmetrical flow in the main microchannel. The two incoming fluid streams form similar flow structures till Re = 80. For the range of the Reynolds number reported in the present work, there is no significant effect on the interface in the simple T-mixer, and hence, there is no influence on mixing performance. The end view of the streamlines is shown at different Reynolds numbers (1, 20, 50 and 80) along with the three-dimensional view (Re = 40). It will be helpful to understand the development of the flow field by visualization and to explain the reason for increasing mixing performance. In the vortex T-mixer, at Re = 1, there is no vortex flow due to very low inertia in the incoming fluid streams. The trajectories of the fluid streams from the two inlet channels are parallel to the walls of the main microchannel. At Re = 20, vortex flow can be visualized, but is not strong enough to make full rotation in the mixing channel. The three-dimensional view of the projected streamlines (for vortex T-mixer at Re = 40) shows that the intensity of the vortex flow is highest near the junction and decreases gradually along the microchannel length. The region affected by the vortex flow increases with the increase in the Reynolds number due to the high inertia force. After a certain distance in the channel, the influence of the vortex flow diminishes.

**Figure 5:**
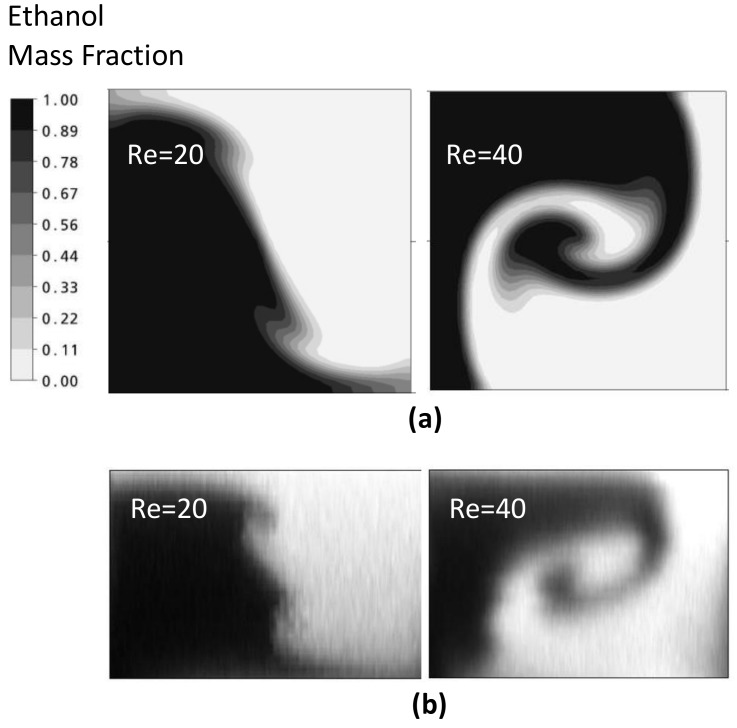
Comparison of the simulation and experimental results for the vortex micro T-mixer: (**a**) numerical results for the mass fraction distribution on y-z plane and (**b**) experimental results for the cross-sectional image [38].

The analysis results for the simple and vortex T-mixer are represented by the mass fraction distributions on the y-z plane (at a fixed axial distance) at various Reynolds numbers for visualizing mixing. The results of numerical and experimental analyses are compared in Figure 5 for Re = 20 and 40. The flow structures are quite similar; even the kinks formed in the numerical simulation at Re = 20 were clearly seen in the cross-sectional image.

**Figure 6:**
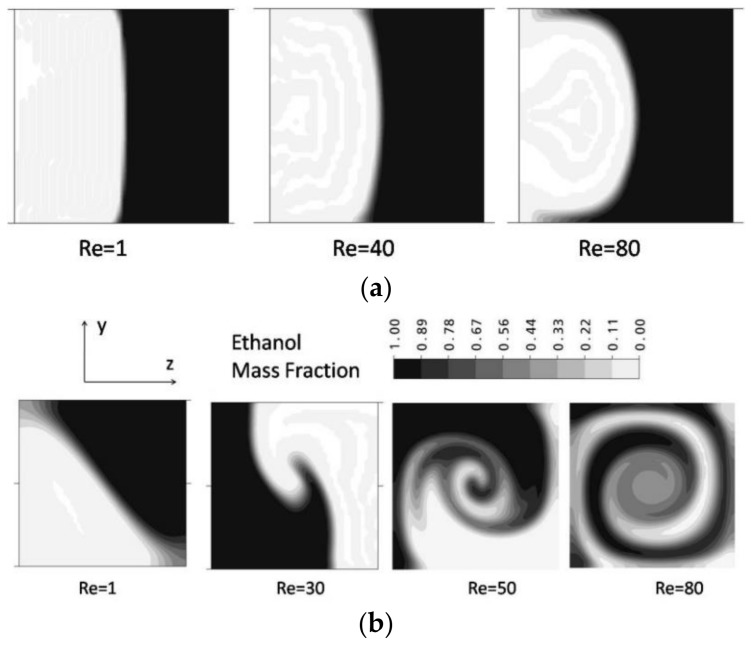
Mass fraction distributions of ethanol on the y-z plane at different Reynolds numbers: (**a**) simple micro T-mixer and (**b**) vortex micro T-mixer.

Figure 6a shows the mass fraction distributions for the simple T-mixer on the y-z plane at Re = 1, 40 and 80. The two fluid streams from the inlet channels form a vertical interface in the middle of the microchannel. There is no significant increase in the interface area of the fluid streams till Re = 80. In the vortex T-mixer (Figure 6b), the mass fraction distributions are shown at Re = 1, 30, 50 and 80. In the vortex T-mixer, at Re = 1, the interface of the fluid streams is diagonally oriented to the cross-section of the mixing channel, and there is no formation of any vortex flow due to low inertia force in the fluid streams. The orientation of the interface is similar at all axial locations in the main mixing channel. At Re = 30, the interface of the fluid streams shows some deformation and reorientation. With increasing Reynolds number, the fluid streams from the two inlets strike with high velocity and create stronger vortex flow (Re = 50 and 80). This type of vortex flow, which effectively stretches the interface area of the fluid streams, is desired for effectively increasing mixing. There is a big difference in the flow structures developed in the simple and vortex T-mixers. Hence, there is no improvement in the mixing performance with the increase in Reynolds number. However, in the vortex T-mixer, there is a significant increase in the interface area due to stretching by vortex flow. Such a type of flow field is desired for effectively increasing the interface of the fluid streams and hence attaining better mixing.

**Figure 7:**
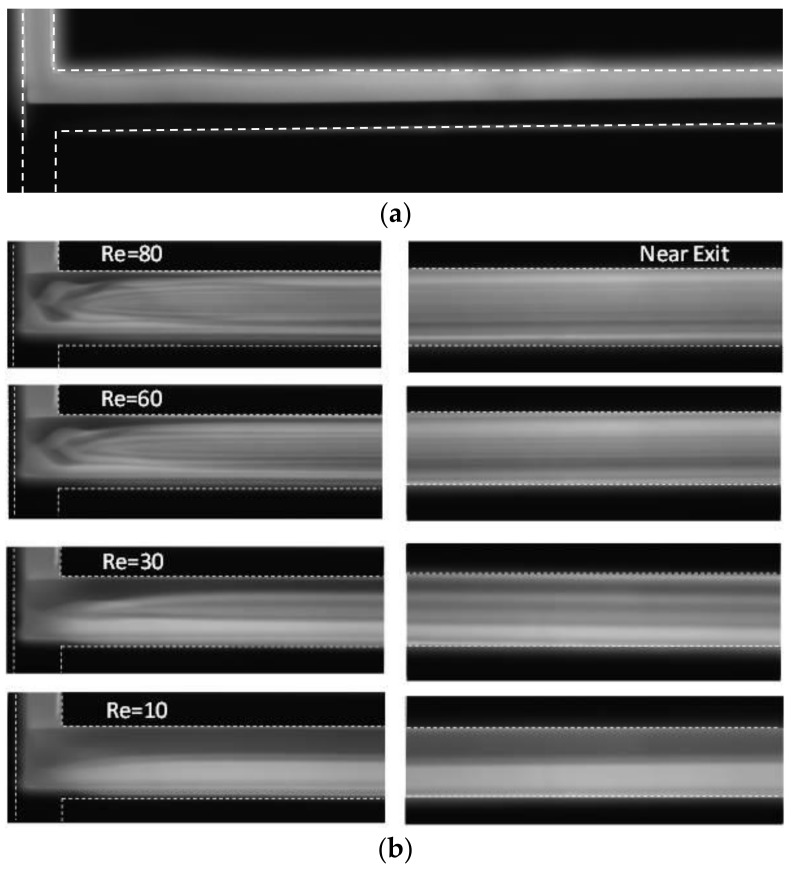
Planar mixing images obtained by experiment for (**a**) the simple T-mixer, Re = 80 and (**b**) the vortex micro T-mixer at different Reynolds numbers near the junction (left) and the exit of the mixing channel (right).

The flow structure and mixing of fluids explained above using the numerical results can be supported by experimental results. Figure 7 shows the planar mixing images obtained by experiment for a simple and a vortex T-mixer at different Reynolds numbers. In the simple T-mixer, the two incoming fluid streams form a clear interface in the middle of the microchannel that exist till the end of the microchannel. The mixing image is presented only for Re = 80, as the flow fields are similar below this Re. On the contrary, the vortex T-mixer shows a varied flow field with the Reynolds number. At Re = 10, a little mixing is observed as the two fluid streams form an interface that exists till the end of the microchannel. The incoming fluid streams are able to make a rotation at Re = 30 and above. However, as the Reynolds number increases, the device shows better mixing performance. At Re = 60 and 80, the distribution of the florescence in the image shows higher uniformity near the exit, showing better mixing.

**Figure 8:**
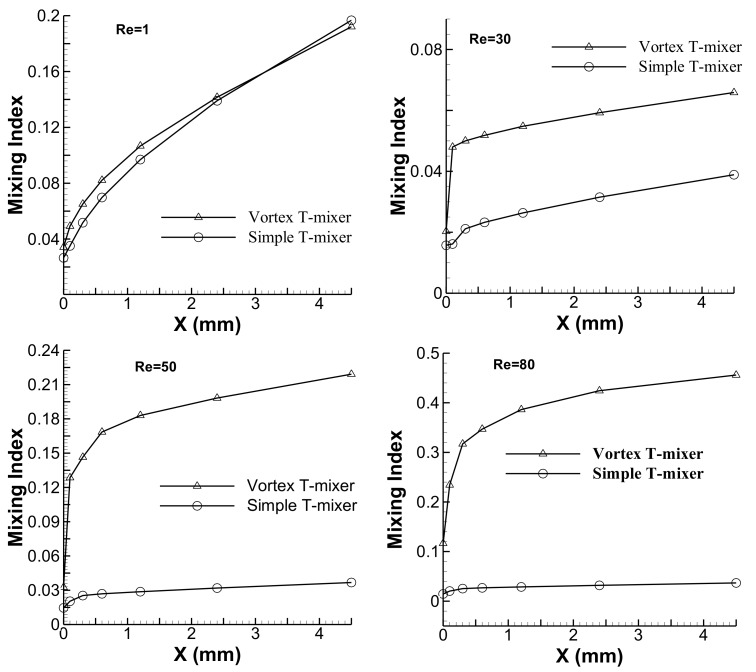
Numerical results for the development of the mixing index along the *x*-axis at various Reynolds numbers for simple and vortex T-mixers with the straight mixing channel.

The mixing index has been evaluated along the *x*-axis at different Reynolds numbers (1, 20, 50 and 80) to quantitatively investigate and compare the mixing performances of the simple and vortex T-mixers (see Figure 8). At Re = 1, both the micromixers show similar mixing characteristics, where the mixing performance gradually increases along the channel length. In the initial section of the main mixing channel, the vortex T-mixer shows slightly higher mixing than the simple T-mixer. This may be due to the large interface area of the fluids streams attained by its diagonal position in the mixing channel (see Figure 5b). However, this difference in mixing vanishes in the later part of the channel. The reason for this is not clear, but may be due to some other effects, like diffusion distance, local residence time of the interface of the fluid streams or other fluid dynamics effects. However, at higher Reynolds numbers, inlet channel modification distinguishably affects the mixing performance. In the vortex T-mixers, the rapid jump in mixing is happening in the initial section of the microchannel, which is due to the formation of the vortex flow. Moreover, the flow has no vortex or the flow becomes simple flow in the remaining part of the mixing channel. In the case of the simple T-mixer, very slow mixing is shown, and there is only a small increase in the mixing index from the T-junction to the end of the main microchannel. The simple T-mixer cannot significantly increase the interface area of the fluid for the reported range of Reynolds number (1 ≤ Re ≤ 80). The difference in the mixing performance between the simple and vortex T-mixers increases as the Reynolds number increases due to an increase in the intensity of the vortex flow caused by the high inertia force of the incoming fluid streams.

### 4.2. Integrating with Serpentine Microchannel

**Figure 9:**
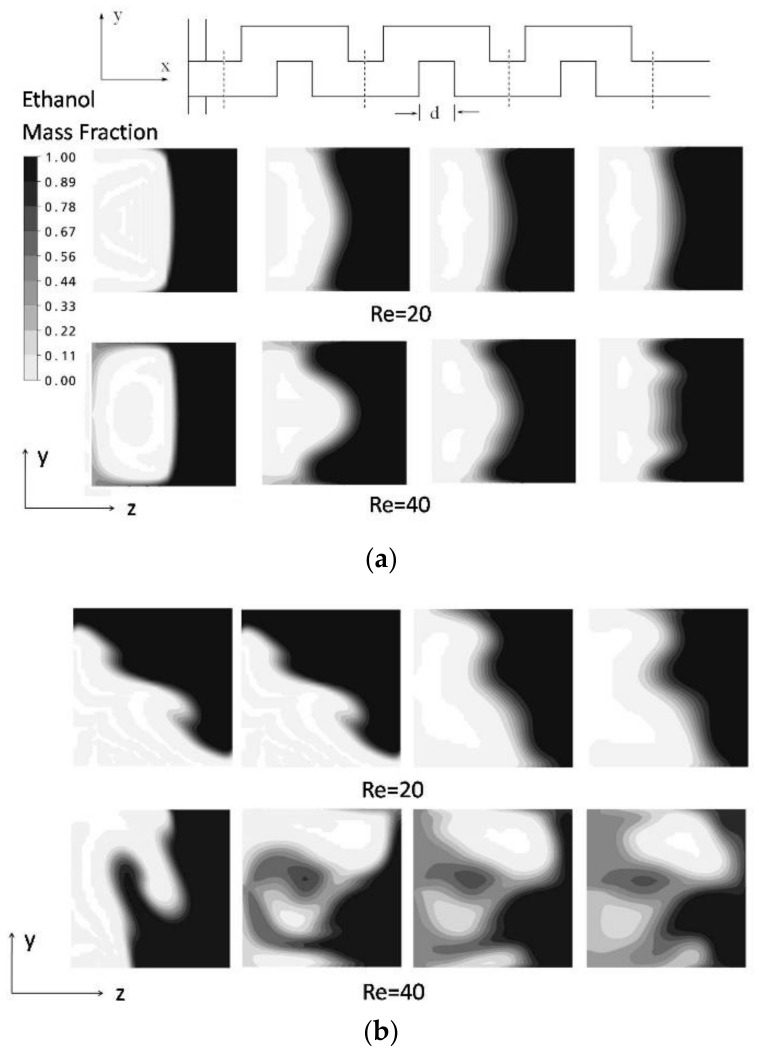
Mass fraction distributions of ethanol on the y-z planes at the initial four axial positions (shown by the dotted lines) in the planar serpentine channel at Re = 20 and 40 integrated with the (**a**) simple micro T-mixer and (**b**) vortex micro T-mixer.

**Figure 10:**
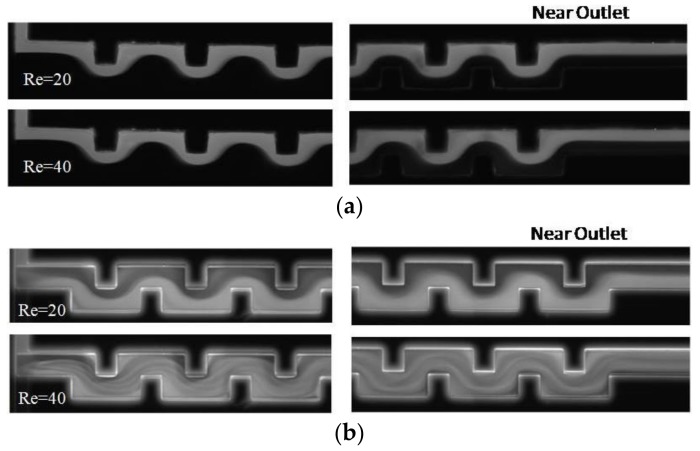
Experimental results. Micrographs for the intensity distribution in serpentine channels (at Re = 20 and 40) integrated with the (**a**) simple T-junction and (**b**) vortex T-junction. In the case of the serpentine channel with the simple T-mixer, the height of the channel is equal to the height of the inlet channels. In such a situation, the heights of the serpentine channel with the simple and vortex T-junctions are 100 µm and 200 µm, respectively.

**Figure 11:**
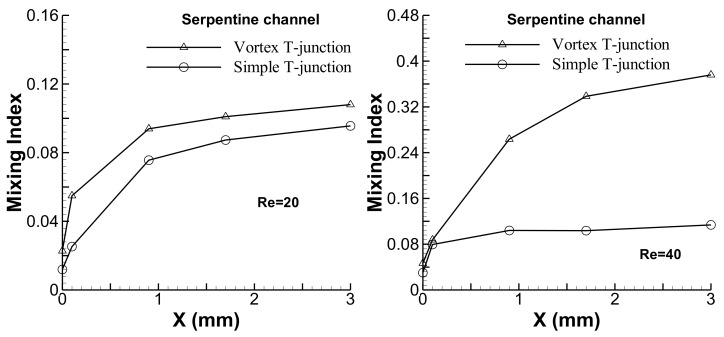
Comparison of the mixing performances of planar serpentine channels integrated with simple and vortex T-junctions.

The mixing performances of the simple and vortex T-junctions have been evaluated and compared by combining them with a planar serpentine channel. The contour plots for the mass fraction distribution on the y-z planes are shown for the serpentine microchannel integrated with the simple and vortex T-junctions at Re = 20 and 40 in Figure 9. In the microchannel with the simple T-junction, the interface of the fluid streams seems very clear and shows only little deformation along the *x*-axis. Better mixing performance can be clearly observed for the microchannel integrated with the vortex T-junction. Experimental results showing the planar mixing images for the simple and vortex T-junctions integrated with the serpentine channel are presented near the junction and exit of the channel at Re = 20 and 40 (see Figure 10). The serpentine channel with the simple T-junction shows very little mixing, and the interface of the fluid streams exists till the end of the channel at Re = 20 and 40. The serpentine microchannel with the vortex T-junction shows poor mixing at Re = 20, but shows better mixing at Re = 40. The mixing was quantified by evaluating the mixing index for the planar serpentine channels integrated with the simple and vortex T-junctions at Re = 20 and 40 (see Figure 11). At these Reynolds numbers, higher mixing performances are shown by the serpentine channel with the vortex T-junction as compared to that with the simple T-junction. The difference in the mixing performance between the vortex and simple T-junctions becomes more pronounced at higher Reynolds numbers.

## 5. Conclusions

A comparative analysis has been carried out on simple and vortex micro T-mixers, both numerically and experimentally. In the vortex T-mixer, the height of the inlet channels is half the height of the main microchannel. The vortex T-mixer creates vortex flow, which increases the interface area of the fluid streams and, hence, increases mixing performance. The mixing performance of the vortex T-mixers is significantly higher than the simple T-mixers. The flow field developed in the simple T-mixer for the reported range of Reynolds numbers (1–80) is not favorable in facilitating rapid mixing. However, in the vortex T-mixer, mixing performance significantly increases in this range of Reynolds numbers. Mixing performances of the simple and vortex T-junctions have been further demonstrated by integrating them with a planar serpentine microchannel at Re = 20 and 40. The planar serpentine microchannel with the vortex T-junction shows higher mixing performance compared to that with the simple T-junction. Therefore, the vortex T-junction can be selected as an inlet junction of all the possible designs of micromixers to enhance the mixing of fluids.
